# Liposomal Delivery of miR-34b-5p Induced Cancer Cell Death in Thyroid Carcinoma

**DOI:** 10.3390/cells7120265

**Published:** 2018-12-11

**Authors:** Hamidreza Maroof, Farhadul Islam, LanFeng Dong, Prabha Ajjikuttira, Vinod Gopalan, Nigel A.J. McMillan, Alfred K. Lam

**Affiliations:** 1 Cancer Molecular Pathology, School of Medicine, Griffith University, Gold Coast, Queensland 4222, Australia; hamidreza.maroof@griffithuni.edu.au (H.M.); farhad_bio83@ru.ac.bd (F.I.); v.gopalan@griffith.edu.au (V.G.); 2 Department of Biochemistry and Molecular Biology, University of Rajshahi, Rajshahi 6205, Bangladesh; 3 School of Medical Science, Griffith University, Southport, Queensland 4222, Australia; l.dong@griffith.edu.au (L.D.); p.ajjikuttira@griffith.edu.au (P.A.); n.mcmillan@griffith.edu.au (N.A.J.M.)

**Keywords:** miR-34b, anaplastic thyroid carcinoma, liposome

## Abstract

This study aims to determine the functional roles of microRNA-34b-5p (miR-34b) in the suppression of anaplastic thyroid carcinoma. We used hydration-of-freeze-dried-matrix (HFDM) formulated liposomes (liposome-loaded miR-34b) for effective delivery of miR-34b to anaplastic thyroid carcinoma in vitro and in vivo. Real time polymerase chain was used to determine the level of miR-34b. Immunocytochemistry, Western blot and ELISA were carried out to determine the effect of this manipulation on VEGF-A expression. In addition, an in vivo xenotransplantation mouse model was used to investigate the functional roles of overexpression of miR-34b in the carcinoma. In anaplastic thyroid carcinoma cells, miR-34b expression was low and significant overexpression (*p* < 0.05) was noted following transfection with liposome-loaded miR-34b. The miR-34b overexpressed thyroid carcinoma cell lines showed reduction in VEGF-A protein expression, decreased cell proliferation, decreased wound healing, reduced cell cycle progression and increased apoptosis (*p* < 0.05). In in vivo experiments, when compared to control groups, smaller tumours formed upon intravenous administration of liposome-loaded miR-34b. To conclude, the current study confirmed the tumour suppressor properties of miR-34b via VEGF-A regulation in anaplastic thyroid carcinoma. In addition, delivery of miR-34b using cationic liposome could be a useful therapeutic strategy for targeting therapy in the carcinoma.

## 1. Introduction

Anaplastic thyroid carcinoma of thyroid, also known as undifferentiated thyroid carcinoma, is one of the most lethal human malignancies, with the most complex genetic make-up in thyroid carcinoma [1]. It is worth noting that papillary thyroid carcinoma, the most common thyroid cancer, can dedifferentiate to anaplastic thyroid carcinoma [2,3]. The current treatment modalities are not effective for this type of thyroid cancer and only a small percentage of patients with carcinoma survive beyond two years [3,4]. Thus, to improve the quality of life of patients with anaplastic thyroid carcinoma, we need an understanding of the carcinogenesis to develop new modes of treatment for this carcinoma. 

In anaplastic thyroid carcinoma, several microRNAs could suppress the progression of the carcinoma in vitro and in vivo [5,6,7]. Micro-34b-5p functions as a potent regulator of angiogenesis and have been reported to be involved in thyroid carcinoma [8,9,10]. 

The most important factor controlling angiogenesis is vascular endothelial growth factor (VEGF). Angiogenesis is important in the pathogenesis of thyroid carcinoma. In thyroid carcinoma, we noted that expression of VEGF correlates with the presence of lymph node metastases and higher risk of cancer recurrence [11,12,13]. In addition, we have documented many functional roles of VEGF in thyroid carcinoma [14,15]. The 3′ UTR (untranslated region) of VEGF contains miR-34b5p binding site [10]. Thus, miR-34b-5p had a suppressive effect on anaplastic thyroid carcinoma via modulation of VEGF-A [8]. 

Many delivery strategies are available to enhance the uptake of miRNA to the target cancer cells. However, many methods have their limitations [16]. One delivery method, the polyethylene glycol (PEG)-ylated lepidic system (liposome), showed effectiveness in targeting cancers [17,18,19]. Wu and colleagues described a novel liposome formulation, the hydration of a freeze-dried matrix (HFDM) [20]. These PEGylated siRNA-loaded lipid particles contain *N*-[1-(2,3-dioleoyloxy)propyl]-*N*,*N*,*N*-trimethylammonium methyl-sulfate (DOTAP), cholesterol, 1,2-dioleoyl-sn-glycero-3-phosphoethanolamine (DOPE) and polyethylene glycol (PEG) 2000-C16Ceramide (50:35:5:10 molar ratio). They could deliver siRNAs effectively to the spleen, lung, liver and cancer cells [20,21,22,23,24,25,26,27,28]. This method can protect miRNA from degradation by reticuloendothelial organs as well as nucleases and other serum proteins. Thus, the miRNA of interest could accumulate in the carcinoma cells both in vitro and in vivo.

In this study, we investigated the biological effects and mechanisms of miR-34b on proliferation and angiogenesis in anaplastic thyroid carcinoma by liposomal delivery of the miR-34b to the carcinoma cells in vitro and in vivo.

## 2. Material and Method

### 2.1. Cell Culture 

The thyroid carcinoma cells used in this study were from cell lines, 8505C (derived from anaplastic thyroid carcinoma), BHT-101 (derived from lymph node with metastatic anaplastic thyroid carcinoma) and Nthy-ori 3-1 (A non-neoplastic thyroid follicular cell line). 8505C and BHT-101 were purchased from Deutsche Sammlung von Mikroorganismen und Zellkulturen GmBH-German Collection of microorganisms and cell cultures (DSMZ, Braunschweig, Germany) and Nthy-ori 3-1 obtained from European Collection of Cell Cultures (ECACC). The 8505C cells were cultured with Roswell Park Memorial Institute medium (RPMI 1640) (Invitrogen, Carlsbad, CA, USA), 2 mM l-glutamine (Invitrogen) supplemented with 10% (*v*/*v*) foetal bovine serum (Invitrogen). The BHT-101 cells were cultured with Dul-becco′s Modified Eagle′s medium (DMEM) (Invitrogen) supplemented with 20% foetal bovine serum (Invitrogen), 0.5% human serum (Sigma, St. Louis, MO, USA), 5 μg/mL human insulin (Sigma) and 2 mM l-glutamine (Invitrogen). The cell lines were authenticated by standard protocol (using multiplex polymerase chain reaction of mini-satellite markers for DNA fingerprinting and identification of short tandem repeats of cell lines and cytogenetics). In addition, the passage number of these cell lines was less than nine. 

### 2.2. HFDM Formulated miRNA-Entrapped PEGylated Lipid Particle 

DOTAP (1,2-dioleoyl-3-trimethylammoniumpropane), DOPE (dioleoylphosphatidylethanolamine), cholesterol, and PEG2000-C16 ceramide (*N*-palmitoyl-sphingosine-1-{succinyl[methoxy(polyethylene glycol)2000]}) were purchased from Avanti Polar Lipids (Alabaster, AL, USA). Hydration of a freeze-dried matrix (HFDM) method was used for the preparation of liposomes as described previously [20]. Briefly, DOTAP, cholesterol, DOPE and PEG2000-C16 ceramide at a molar ratio of 50:35:5:10, were mixed with mature miR-34b mimic sequence (guide strand) 5′-UAGGCAGUGUCAUUAGCUGAUUG-3′ (Qiagen, Hilden, Germay) at nitrogen: phosphate (N:P) ratio of 4 in sucrose-containing water/tert-butanol (1:1 *v*/*v*) co-solvent system. The resultant formulation was then snap frozen and freeze-dried overnight in a freeze-dryer (ALPHA 1–2 LDplus) (Martin Christ, Osterode am Harz, Germany) at −80 °C and <0.1 mbar. Sterile water was used for hydration of freeze-dried matrix immediately before use. The final product contained 40 µg miR-34b in 300 µL of isotonic sucrose solution. 

### 2.3. Nanoparticle Characterization

Zetasizer Nano ZS (Malvern Instruments, Malvern, UK) was used for measurement of size, polydispersity index, miR-34b entrapment efficiently and zeta potential of the resultant liposomes following appropriate dilution in distilled water. Measurements were performed at room temperature. Two size measurements were performed with 10 runs per measurement undertaken.

### 2.4. Liposome-Loaded miR-34b Transfection

The anaplastic thyroid cancer cell lines were transfected with liposome-loaded miR-34b and with liposome-loaded miR-1, a non-targeting control, positive control (miR-1) (Qiagen) and an empty liposome, immediately after being seeded at a density of 20 × 104 cells/well in 6 well-plate. To each well, we added one ml of 5 nM liposome-entrapped miR-34b suspended in antibiotic-free complete media. After this, the cells were maintained at 37 °C at 5% carbon dioxide and monitored for 48 h. The same protocol was performed for liposome-loaded miR-1 and empty liposome transfection.

### 2.5. Quantification of miR-34b Expression

Total miRNA from cells was extracted using NucleoSpin^®^ miRNA Kit (MACHEREY-NAGEL, Duren, Germany) with a DNase additional step. The purity of miRNA was examined by checking the optical density (OD), using a nanodrop spectrophotometer. Then, cDNA was synthesised using miScript II RT Kit (Qiagen, Venlo, The Netherlands) according to the manufacturer instructions.

The miR-34b expression level was quantified by real-time quantitative polymerase chain reaction (qRT-PCR) using Hs_miR-34b *_2 miScript Primer Assay (Qiagen) following previously published protocol10. Samples were normalised using the housekeeping gene *RNU6B RNA* (Hs_ RNU6B_2 miScript Primer Assay, Qiagen).

Amplification, detection and analysis were performed with a QuantStudio™ 6 Flex Real-time PCR system (Applied Biosystems, Foster City, CA, USA). Real-time PCR amplifications were performed in 20 μL reaction volume consisting of 10 μL QuantiTect SYBR Green PCR Master Mix (Qiagen), 1 μL miScript Primer Assay (Qiagen), 1 μL of miScript Universal Primer (Qiagen), and 5 μL of cDNA template at 2 ng/μL stock and 3 μL RNase-free water. All qRT-PCR reactions were carried out in triplicates with a non-template control as previously published protocol [29]. Expression of miR-34b was presented as the ratio between miR-34b and RNU6B. The 2-∆∆ct method was used to calculate the fold changes of miRNA in each sample group. Less than 0.5-fold differences were considered as low expression. Fold changes between 0.5 and 2 were considered as normal expression, whereas fold changes of more than 2 were considered as high expression. 

### 2.6. Western Blot Analysis for VEGF-A in Anaplastic Thyroid Carcinoma Cells 

The transfected thyroid cancer cells were lysed in Cell Lysis Buffer NP40 (50 mM Tris, pH 7.4, 250 mM NaCl, 5 mM EDTA, 50 mM NaF, 1 mM Na3VO4, 1% Nonidet P40, 0.02% NaN3) (Invitrogen) supplemented with protease inhibitor cocktail (Sigma), phenylmethanesulfonyl fluoride solution (PMSF) (Sigma) and phosphatase inhibitor cocktail (Cell Signaling, Danvers, MA, USA). Then, whole protein lysates were quantified using the Macherey-Nagel protein assay kit (MACHEREY-NAGEL). Equal quantities of 25 µg protein samples were run on a 4–15% precast polyacrylamide gel (Mini-PROTEAN^®^ TGX TM Precast Gel, Bio-Rad, Hercules, CA, USA). After separation, the proteins were transferred to the polyvinylidene difluoride (PVDF) membrane. Then, blocking was performed with 5% non-fat milk *w*/*v* in TBST (Tris-buffered saline Tween 20: 120 mmol/L Tris–HCl, pH 7.4, 150 mmol/L sodium chloride, and 0.05% Tween 20) for 2 hours at room temperature. After blocking, the membrane was incubated with anti-VEGF-A (Sc-152), 1:300 dilution and anti- β-actin (Sc-4778), 1:5000 dilution (Santa Cruz Biotechnology, Dallas, TX, USA) overnight at 4 °C. According to the manufacturer’s protocol, blots were washed five times with TBST. Then, they were incubated for 2 h with horseradish peroxidase (HRP)-conjugated secondary antibody (1:5000 dilution) (Santa Cruz Biotechnology) at room temperature. The blots were then developed using Clarity™ Western ECL Blotting Substrate kit (Bio-Rad). They were visualised by ChemiDoc-MP Imaging System (Bio-Rad) and analysed with ImageJ software (National Institutes of Health, Bethesda, MD, USA).

### 2.7. Enzyme-Linked Immunosorbent (ELISA) Assay

The thyroid cancer cells were incubated in low-serum media (DMEM: F12 with 1% foetal bovine serum to preserve VEGF stability) and conditioned media collected after two days for the analysis of VEGF secretion levels as previously described [7]. The cells with liposome-loaded miR-1 transfection and empty liposome were set as controls of liposome-loaded miR-34b treatment group. The concentration of secreted VEGF was measured using a Novex Human VEGF ELISA kit (Life Technologies, Carlsbad, CA, USA) following manufacturer instructions.

### 2.8. Cell Proliferation Assay 

Cell Counting Kit-8 (CCK-8) (Sigma) reagents are used to evaluate the cell viability. The cell densities of 1 × 104 cells/well were seeded in a 96-well tissue culture plate (Becton Dickson and Company, Franklin Lakes, NJ, USA). Cell proliferation with liposome-loaded miR-1 transfection and empty liposome were set as controls of liposome-loaded miR-34b treatment group. On days one to three after the initial seeding, the proliferation of the cells was determined with CCK-8 following manufacturer instructions.

### 2.9. FACS Analysis for Cell Cycle Distribution 

The thyroid cancer cells were fixed with cold 70% ethanol for one hour as reported recently [30]. Briefly, after washing with cold phosphate-buffered saline (PBS), 5 µL of RNase A (10 mg/mL) was added to the cells and incubated for one hour at 37 °C. Finally, 10 µL of propidium iodide solution (1 mg/mL) was added to the cell suspension. Cell cycle distribution was then analysed by flow cytometry using FACS analysis (BD FACSCalibur, BD Biosciences, San Jose, CA, USA). Finally, the percentage of cells in different phases of cell cycle was determined by FlowJo single-cell analysis software (Ashland, OR, USA).

### 2.10. Quantitative Apoptosis Assay

Apoptosis assay was performed to measure the percentage of apoptotic cells using a Membrane Permeability/Dead Cell Apoptosis Kit (Invitrogen). After 48 h of transfection, the thyroid carcinoma cells were harvested and washed twice with ice-cold PBS and resuspended at the 25 × 10^4^ cells/mL in PBS. For staining, 1 µL Annexin V and 1µL PI were added and kept in the dark for 20 min at room temperature. After staining, flow cytometry was performed for the quantification of apoptotic cells using FlowJo single-cell analysis software.

### 2.11. Wound Healing Assay 

To determine the migration capacity of thyroid carcinoma cells after transfection with liposome-loaded miR-34b, a wound healing assay was performed. The carcinoma cells were cultured in six-well plates. Transfection was performed at a final concentration of 5 nM when cells grew up to 70% confluency as a monolayer. After 48 h, scratches were made in liposome-loaded miR-34b, liposome-loaded miR-1 treated cells and empty liposome treated cells with a 200-mL pipette tip across the centre of culture plates. Media was added to the culture after the cells were washed three times with PBS. Images were then taken under an inverted microscope at 0, 24, 48 and 72 h after the wounding. The mobility of cells in different days was measured and compared with ImageJ 1.48 software.

#### In Vivo Study

All the animal experiments performed in this study were in accordance with the ethical standards of Griffith University (MED/01/17/AEC) as well as 1964 Helsinki declaration and its later amendments comparable to the ethical standards.

Subcutaneous tumours from the anaplastic thyroid carcinoma cells (BHT-101) were established in female NU/NU nude mouse of 6–8 weeks old (Animal Resource Centre, Perth, WA, Australia). Two million carcinoma cells suspended in 100 μL of sterile PBS were injected subcutaneously into right and left sides of the abdomen of each mouse. We observed the growth of the tumours daily. Tumours in the mice were detectable by palpation after one week of implantation. We allowed the tumours to grow to 28 days when progressive growth established. The size of the weight tumours were measured using calliper and scale from the day when tumours first being detected from palpation until the end of the experiment. Tumour establishment and progression were monitored at days 7, 14, 21, and 28 using callipers during the experiment. After 28 days, the mice were injected intravenously with liposome-loaded miR-34b and with liposome-loaded miR-1 (2 mg/kg of microRNAs-40 μg per dose per mouse) and empty liposome on day, 29, 33, 37, 41, 45, 49 and 53. All formulation was prepared at N: P ratio of 4. On day 56, the mice were euthanised and autopsies were performed. The tumours were removed from the mice. Tumour volume was assessed by measurement with callipers using the following formula: tumour volume = (length × width × height)/0.5236 and 6 mice were used per treatment group (*n* = 6) [31]. All the organs in the chest and abdomen were examined. The tumours as well as the lung, liver and kidney from each mice were formalin fixed and embedded in paraffin. A portion of each a tumour was snap frozen at –80 °C for downstream (miRNA and protein) analysis.

### 2.12. Histological Analysis and Proliferative Marker 

Hematoxylin and eosin staining of the sections were taken from the paraffin blocks of mice according to a previously published protocol [32]. The morphology of tumours was analysed and confirmed by a pathologist. Formalin-fixed, paraffin-embedded tumour tissue sections were used to investigate the expression level of the proliferative marker (Ki-67 protein). Immunostaining was performed by the peroxidase-indirect-polymer method. Tumour tissue sections were deparaffinized, rehydrated and subjected to epitope antigen retrieval 2 (ER2) (20 min at 94 °C) (Leica Biosystems, AR9640, Biosystems, Wetzlar, Germany) with target retrieval solution high pH (50x) Dako EnvisionTM Flex (Agilent, Santa Clara, CA, USA) in a pre-treatment module PTlink (Dako, Model PT 10130). Primary monoclonal mouse antibody anti-human Ki-67 (Clone MIB-1, M7240; Dako) at 1:50 was used. Immunohistochemistry was performed using an automated stainer (BOND-III, Leica, Biosystems, Wetzlar, Germany) by the peroxidase-indirect- polymer method (K8000, Dako) for Ki-67. The positive control was histological sections of tonsil on every tested section. For negative controls, the primary antibody was omitted during the staining. Proliferation index was expressed as a percentage of Ki-67-positive cells. Image J software was used for quantitation of Ki-67 positive cells [7]. 

### 2.13. miR-34b and VEGF-A mRNA Studies in Mice Xenografts

For extraction of RNA and miRNA from cancer tissues from the mice, a cryostat (Leica Biosystems, Mt Waverley, VIC, Australia) was used to section cancer tissues. Tissues were sectioned and RNA, miRNA extraction was performed as previously described [33]. Briefly, RNA and miRNA extraction from tissues were performed with Qiagen miRNeasy FFPE Kits (Qiagen Pty. Ltd., Hilden, NRW, Germany). The purity of RNA and miRNA was examined by checking the optical density (OD) using a Nanodrop spectrophotometer. The purified RNA and miRNAs were converted to cDNA using miScript Reverse Transcription kits (Qiagen) according to the manufacturer’s instructions. The miR-34b expression level was quantified by real-time quantitative polymerase chain reaction (qRT-PCR) as described above. 

### 2.14. Western Blot Analysis for VEGF-A in Mice Xenografts 

Fresh frozen cancer tissues collected from the mice at dissection were processed for Western blot similar to our published protocol [34]. In brief, total proteins were extracted from fresh frozen tissue samples with lysis buffer (Bio-Rad, Gladesville, NSW, Australia). These were followed by homogenisation and quantitation by absorbance spectrometry. Afterwards, total protein (30 μg) was separated by 15% SDS-PAGE (Bio-Rad) and transferred to nitrocellulose membranes (Bio-Rad). The membrane was then developed to detect protein bands according to our previously published protocol [10]. Expression of VEGF-A protein was quantified and normalised to β-actin with the ImageJ software.

### 2.15. Data Analysis

Results were analysed using GraphPad Prism 7.0 (Graph Pad Software, San Diego, CA, USA) and were expressed as means ± SD (standard deviation). All the in vitro experiments were performed at least three times. Tumour volume from the mice xenograft model was analysed using a two-tailed paired t-test. Statistical comparisons between groups were conducted using one-way ANOVA. A *p* value of <0.05 was considered statistically significant and individual *p*-values was shown in the figures.

## 3. Results 

### 3.1. Characterization of Lipid Nanoparticles Entrapped miR-34b 

Liposome formation of miR-34b (liposome-loaded miR-34b) by the hydration of a freeze-dried matrix (HFDM) method could effectively deliver miR-34b to the thyroid carcinoma cells (Table 1). After rehydration, the characteristics features such as size, zeta potential, polydispersity index of the resulting lipid particles and the entrapment efficiency of miR-34b were measured. The results indicated that the prepared liposome-loaded miR-34b liposomes had favourable characteristics for effective delivery of miR-34b with high entrapment efficiency and size below 200 nm.

### 3.2. Liposome-Loaded miR-34b Overexpression Caused Downregulation of VEGF-A Protein 

**Figure 1:**
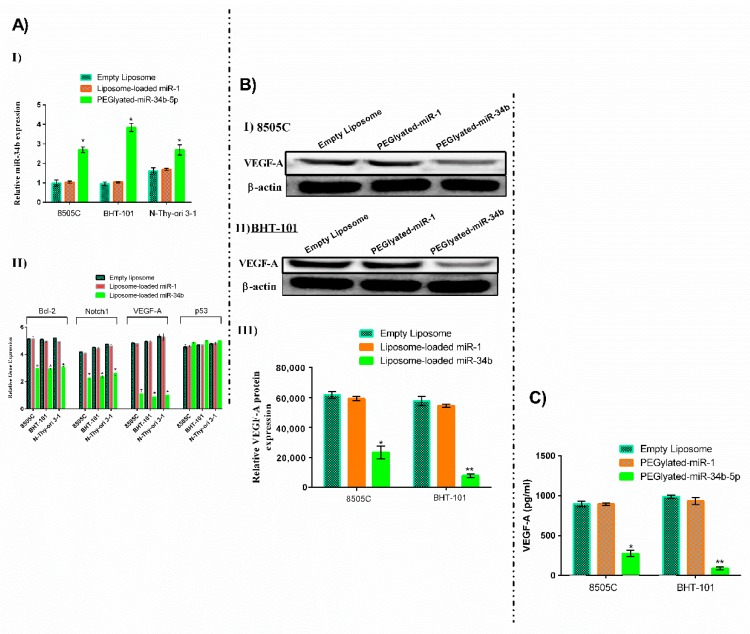
The effects of miR-34b overexpression on VEGF-A expression in 8505C, BHT-101 and N-thy-ori 3-1 cells. (**A**) (**I**) After two days, relative miR-34b expression after transfection with liposome-loaded miR-34b, liposome-loaded miR-1 and empty liposome, detected by qRT-PCR. Liposome-loaded miR-34b transfection led to overexpression of miR-34b in anaplastic thyroid carcinoma cells. miR-34b expression level was significantly increased after transfection with liposome-loaded miR-34b when compared to liposome-loaded miR-1, empty liposome transfected cells and N-thy-ori 3-1 (A non-neoplastic thyroid follicular cell line) (**II**) relative gene expression after transfection with liposome-loaded miR-34b, liposome-loaded miR-1 and empty liposome, detected by qRT-PCR. No significant changes in p53 observed and VEGF-A in more downregulated in comparison with other genes. (**B**) miR-34b overexpression alters VEGF-A expression in anaplastic thyroid carcinoma cells. Western blot analysis showed the decreased expression levels of angiogenesis related protein VEGF-A, after overexpression of miR-34b. VEGF-A expression were changed upon liposome-loaded miR-34b transfection in the anaplastic thyroid carcinoma cells: 8505C (**I**) and BHT-101 (**II**). VEGF-A protein expression was downregulated in anaplastic thyroid carcinoma cells (8505C and BHT-101) when compared to liposome-loaded miR-1 and empty liposome transfected cells (**III**). (**C**) Overexpression of miR-34b inhibits VEGF expression in thyroid cancer cell lines supernatant. Protein levels of VEGF-A of conditioned media measured by ELISA, showing significant reduction of secreted VEGF-A after liposome-loaded miR-34b, liposome-loaded miR-1 and empty liposome transfection in 8505C and BHT-101 cell lines when compared to the control groups. Data are presented as mean ± SD from three independent tests. Level of significance, * *p* < 0.05, ** *p* < 0.01 when compared to the control groups.

Exogenous overexpression of miR-34b was carried out in anaplastic thyroid carcinoma to investigate its specific regulatory function on VEGF-A expression. Cells treated with the liposome-loaded miR-34b showed significant (*p* < 0.05) overexpression of miR-34b levels (shown as a ratio of expression) when compared to the liposome-loaded miR-1 and empty liposome transfected cells (Figure 1(AI)) and no significant changes observed for p53 (Figure 1(AII)). 

As shown in Figure 1B, the liposome-loaded miR-34b treatment resulted in a significant decrease in VEGF-A protein expression in anaplastic thyroid carcinoma cells in 8505C (Figure 1(BI,III)) (*p* < 0.05) and BHT-101 (Figure 1B(II,III)) (*p* < 0.01). The liposome-loaded miR-1 and empty liposome treated cells showed no changes. A similar trend was noted in protein expression of VEGF-A in the cell culture supernatant of the carcinoma cell lines using ELISA (Figure 1C). The serum VEGF-A protein expression in the transfected group was decreased remarkably and the decrease in expression level was more in BHT-101 cells (*p* < 0.01) than in 8505C cells (*p* < 0.05) when compared to liposome-loaded miR-1 and the empty liposome control.

### 3.3. Tumour Suppressor Properties of Liposome-Loaded miR-34b In Vitro 

**Figure 2:**
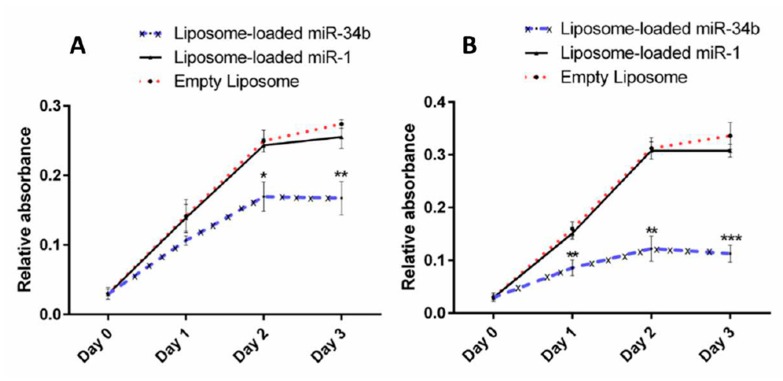
The effect of miR-34b upregulation on proliferation of thyroid carcinoma cells. Overexpression of miR-34b significantly inhibited proliferation of cells in 8505C (**A**) and BHT-101 (**B**). Both anaplastic thyroid carcinoma cell lines treated with liposome-loaded miR-34b showed notable reduced cell proliferation when compared to liposome-loaded miR-1 and empty liposome transfected groups on different days after transfection. The results are shown as mean ± SD from three independent tests. Level of significance, * *p* < 0.05, ** *p* < 0.01, *** *p* < 0.001 when compared to the control groups.

Thyroid carcinoma cells treated with liposome-loaded miR-34b demonstrated a significant decrease in cell proliferation when compared to liposome-loaded miR-1 and empty liposome-transfected cells (Figure 2). Both thyroid carcinoma cell lines showed a notable (*p* < 0.001) decrease in cell proliferation on day 3 of transfection (Figure 2A,B), with the BHT-101 cells more significantly supressed, consistent with the lower levels of VEGF-A observed in these cells upon miR34b overexpression. 

**Figure 3:**
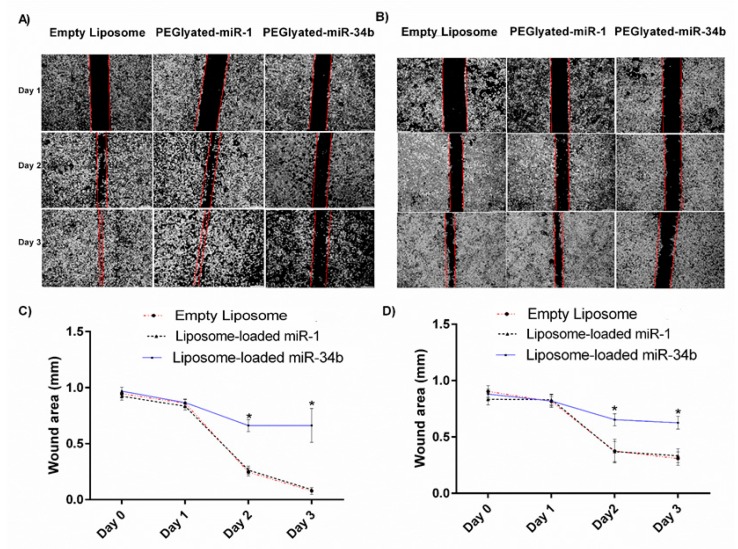
The influence of miR-34b overexpression in wound healing potential of thyroid carcinoma cells. The migration capacity of anaplastic thyroid carcinoma (8505C cells) significantly decreased after day 3 of transfection with miR-34b (**A**) and wound healed faster in liposome-loaded miR-1 and empty liposome transfected control groups in comparison to liposome-loaded miR-34b transfected group (**C**). A similar trend was observed in anaplastic thyroid carcinoma cells - BHT-101 (**B**). The wound healed slower in liposome-loaded miR-34b treated compared to liposome-loaded miR-1 and empty liposome transfected control group (**D**). Wound areas of all experimental cell types on different days (day 0 to day 3) were recorded from three independent measurements and shown as mean ± SD. Level of significance, * *p* < 0.05, when compared to the control groups.

Likewise, the wound healing assay showed that 8505C (Figure 3A,C) and BHT-101 (Figure 3B,D) cells transfected with liposome-loaded miR-34b migrated significantly more slowly than cells transfected with liposome-loaded miR-1 or empty liposomes (*p* < 0.05) (Figure 3). 

### 3.4. The Suppressive Role of miR-34b in Apoptosis and Cell Cycle Distribution 

**Figure 4:**
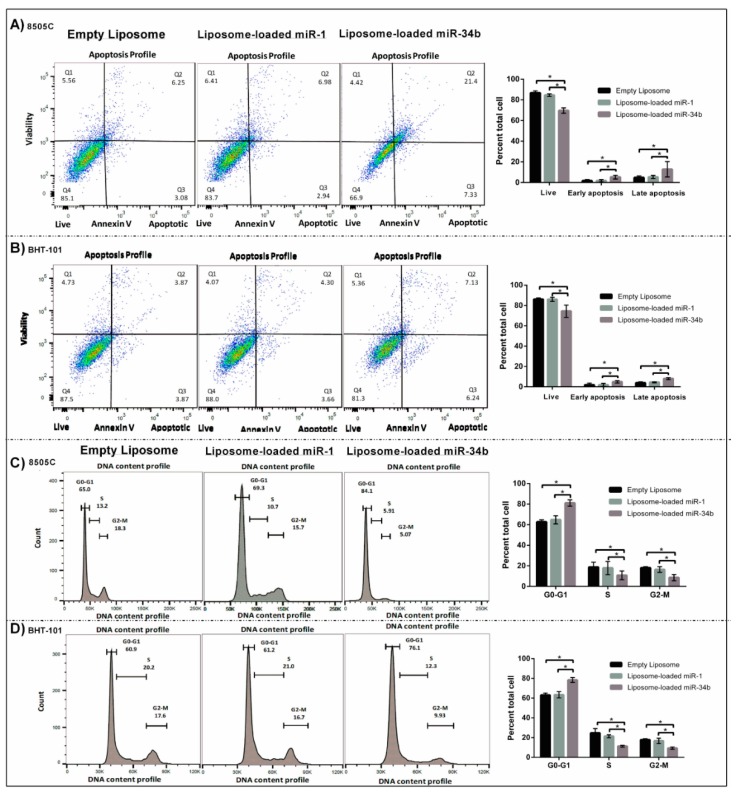
Effect of miR-34b overexpression on apoptosis and cell cycle kinetics of thyroid carcinoma cells. Thyroid cancer cell lines transfected with liposome-loaded miR-34b liposome-loaded miR-1 and empty liposome for 48 h. Apoptosis level of 8505C (**A**) and BHT-101 (**B**) were determined using Annexin V/propidium iodide (PI) staining and flow cytometry analysis. In both cancer cell lines, miR-34b overexpression decreased the total number of apoptotic cells in early and late apoptosis phase. Data were shown as mean ± SD of three independent experiments and represent the percent of AnnexinV-positive cells with miR-34b-5p related to liposome-loaded miR-1 and empty liposome treatment groups. The percentage of dead cells (Q1; upper left quadrant), live cells (Q4; lower left quadrant), late apoptosis cells (Q2; PI+/Annexin V+; upper right quadrant) and early apoptosis cells (Q3; PI-/Annexin V+; lower right quadrant) were indicated. Cell cycle analysis revealed that miR-34b overexpression caused significant accumulations of cancer cells of 8505C (**C**) and BHT-101 (**D**) cells in G0-G1 phase after 48 h of transfection with liposome-loaded miR-34b when compared to liposome-loaded miR-1 and empty liposome transfected control group. Nuclei of the cells were stained with propidium iodide (PI) solution and analysed for DNA content by flow cytometry. Data were shown as mean ± SD of three independent experiments and represent the percentage of cells in different phases of the cell cycle with liposome-loaded miR-34b related to liposome-loaded miR-1 and empty liposome treatment. Flow cytometry results indicated the cell number increased in G0-G1 phase and decreased in S and G2-M phases when compared with liposome-loaded miR-1 and empty liposome treatment groups. Asterisks indicate statistically significant differences (* *p* < 0.05, Student’s *t*-test) when compared to control cells.

Following the noticeable reduction in cell proliferation of thyroid carcinoma cells, apoptosis and cell cycle assays were performed to further examine the mechanism of miR-34b in cancer progression (Figure 4). Increased induction of apoptosis was noted in thyroid carcinoma cells treated with liposome-loaded miR-34b when compared to liposome-loaded miR-1 and empty liposome-transfected control. The percentage of early and late apoptotic event were significantly increased in the 8505C cells (11.6 ± 3.72) (Figure 4A) after two days of transfection with liposome-loaded miR-34b, compared with liposome-loaded miR-1 and the empty liposome-transfected control group (*p* < 0.05). Similarly, in BHT-101 cells, the early and late apoptotic features were significantly (*p* < 0.05) increased after transfection with liposome-loaded miR-34b (12.1 ± 1.30) when compared to liposome-loaded miR-1 and the empty liposome-transfected control group (Figure 4B). In addition, the introduction of liposome-loaded miR-34b into the 8505C cells (Figure 4C), exhibited a significant accumulation of cells in the G0-G1 phase (18.3 ± 0.60) and a substantial drop in S and G2-M phase after 48 h (*p* < 0.05). The BHT-101 cells also expressed a similar trend of cell cycle changes following miR-34b transfection (*p* < 0.05).

### 3.5. In vivo Confirmation of miR-34b Tumour Suppressor Properties

**Figure 5:**
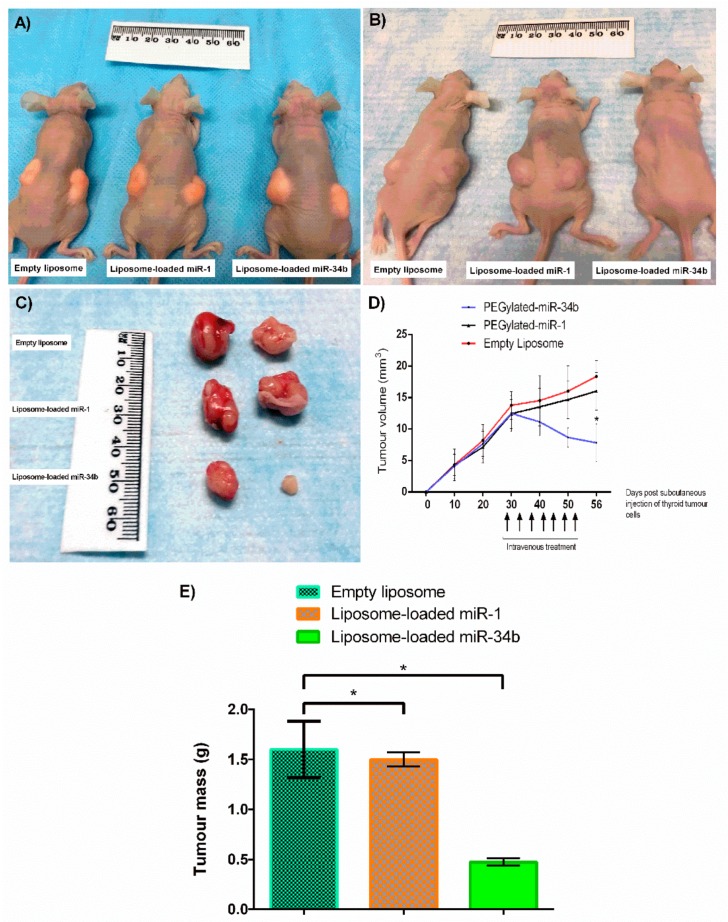
Inhibition of subcutaneous thyroid tumour growth in xenograft nude mouse by liposome-loaded miR-34b. The subcutaneous injection of anaplastic thyroid carcinoma cells (BHT-101) in mice, formed tumour after 28 days (**A**). All treatments were administrated on days 29, 33, 37, 41, 45, 49 and 53, after tumour cell inoculation and 40 μg miRNA was used per dose. Mice that were intravenously injected with liposome-loaded miR-1 and empty liposome, generated larger tumour size, whereas smaller tumour observed in mice that received liposome-loaded miR-34b (**B**). Mice treated with liposome-loaded miR-34b had significant reduced tumour volume (mm^3^) when compared to mice treated with liposome-loaded miR-1 and empty liposome (*n* = 6) (**C** and **D**). Tumours were measured by calliper on subsequent days post initial subcutaneous (s.c.) injection of tumour cells. Results are shown as mean ± SD. Level of significance, * *p* < 0.05, when compared to the control groups. At the beginning of the in vivo study, the weight of mice in liposome-loaded miR-34b, liposome-loaded miR-1 and empty liposome treated group were 20.8 ± 0.04, 21.9 ± 0.1, 22.0 ± 0.09 gram respectively. After 56 days of observation, the weight of mice in liposome-loaded miR-34b treated group was 23 ± 2.0 gram whereas in liposome-loaded miR-1 and empty liposome treated group were 24.8 ± 0.6 and 23.8 ± 0.4 gram, respectively. There was no statistical difference in mouse body weight between the treated and non-treated group. The weight of tumours measured and were significantly lower in liposome-loaded miR-34b compared to liposome-loaded miR-1 and empty liposome treated group (**E**). This indicates that there is no toxicity in treated and non-treated groups.

In our xenograft mouse model, the tumours were noted at one week by palpation. The tumours grew to an approximately 20 mm diameter each 28 days after carcinoma cell implantation (Figure 5A). Subsequently, mice were treated with liposome-loaded miR-34b, liposome-loaded miR-1 or the empty liposome every 4 days for a further 28 days. The veins of the mice were injected on both sides to make multiple injections possible in the experimental period. The tumours in the mice that received liposome-loaded miR-34b, started to shrink (Figure 5B). The volume of the tumours was significantly decreased in the liposome-loaded miR-34b group (7 ± 1.2 mm^3^) when compared with those of liposome-loaded miR-1 (16 ± 0.9 mm^3^) and the empty liposome (18 ± 0.6 mm^3^) group at the end of this study (*p* < 0.05) (Figure 5C,D). At the beginning of the in vivo study, the weight of mice in liposome-loaded miR-34b, liposome-loaded miR-1 and empty liposome treated group were 20.8 ± 0.04, 21.9 ± 0.1, 22.0 ± 0.09 gram respectively. The weight of tumours measured and were significantly lower in liposome-loaded miR-34b compared to liposome-loaded miR-1 and empty liposome treated group (Figure 5E). At autopsy, no carcinoma was present in the other organs of the mice. 

On microscopic examination, no carcinoma present in the lungs, liver and kidneys in each animal. When comparing the control and tested groups, there was no difference in inflammation, necrosis or other significant changes in these organs. 

**Figure 6:**
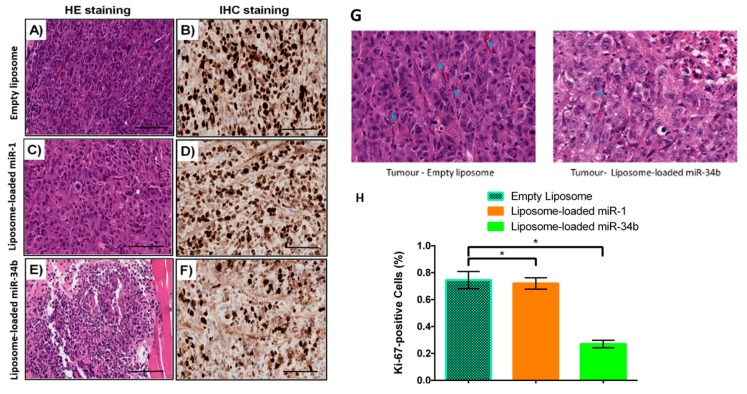
Liposome-loaded miR-34b decreases proliferation in xenograft nude mouse tumour. Tumour xenografts obtained by subcutaneous injection of anaplastic thyroid carcinoma (BHT-101) were formalin fixed and paraffin embedded. 3μm-thick sections were cut for histological and Ki-67 immunohistochemistry analysis. Photographs of the hematoxylin and eosin-stained tissue sections of anaplastic thyroid carcinoma on day 56 after seven intravenous injections of liposome-loaded miR-34b, liposome-loaded miR-1 and empty liposome. 40μg miRNA was used per dose (**A**,**C**,**E**). The carcinoma in the liposome-loaded miR-34b (**E**) was less cellular, less mitotic active and with lower nuclear pleomorphism than other 2 groups (**A**,**C**). The empty liposome treated tumour tissue shows frequent mitosis and more vessels (marked by stars) when compared to the liposome loaded-miR-34b treated tumour (**G**). The treated tumour shows prominent necrosis. Analysis of cell proliferation by Ki-67 immunostaining in tissue sections of thyroid carcinoma. Representative images of Ki-67 immunostaining (40×) (**B**,**D**,**F**), and percentage of Ki-67-positive cells (**H**) in thyroid carcinoma. Higher Ki-67 proliferative index were formed in mice receiving liposome-loaded miR-1 and empty liposome (**B**,**D**) whereas lower proliferative index was generated in mice treated with liposome-loaded miR-34b (**F**). Results are expressed as mean ± SD. Level of significance, * *p* < 0.05, when compared to the control groups. Bar represents 250 μm (**A**–**F**).

The tumours in the liposome-loaded miR-34b treated group showed lower cellularity, mitotic figures solid growth as well as increased in degeneration and necrosis when compared with the control liposome-loaded miR-1 group and the empty liposome groups (Figure 6A,C,E). The tumours from the liposome-loaded miR-34b treated group had less vascular stroma on histological examination (6G). In the liposome-loaded miR-34b treated group, no cancer was detected in one of the injected site of the mice.

**Figure 7:**
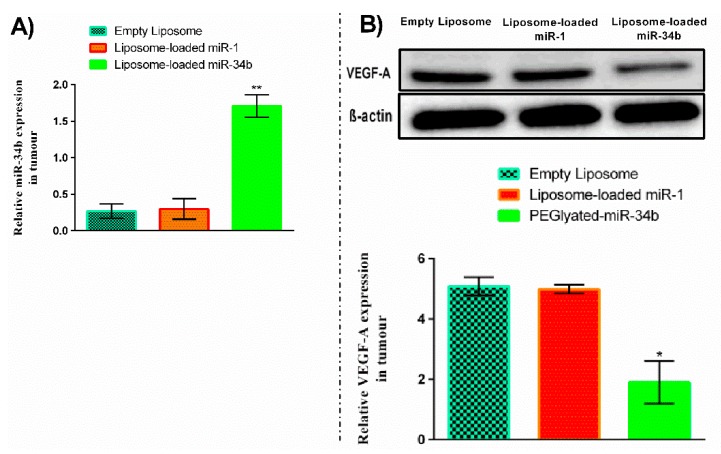
Liposome-loaded miR-34b increases the expression level of miR-34b and decreases VEGF-A protein expression level in xenograft nude mouse tumour. (**A**) Relative miR-34b expression after intravenous injection of liposome-loaded miR-34b, liposome-loaded miR-1 and empty liposome, detected by qRT-PCR (*n* = 6). miR-34b expression level was significantly increased after intravenous injection of liposome-loaded miR-34b in mice when compared to mice injected with liposome-loaded miR-1 and empty liposome. (**B**) VEGF-A protein expression level also was significantly decreased after intravenous injection of liposome-loaded miR-34b in xenograft nude mouse tumour when compared to mice receiving liposome-loaded miR-1 and empty liposome. Data are presented as mean ± SD with *n* = 6. Level of significance, * *p* < 0.05, ** *p* < 0.01 when compared to the control groups.

The proliferative activities of the tumour in the mice were studied by the percentages of nuclei of carcinoma cells stained positive for Ki-67. The fraction of proliferating cells is significantly decreased in liposome-loaded miR-34b when compared to empty liposome treated group (*p* < 0.05) (Figure 6B,D,F,H). The expression level of miR-34b remarkably increased (*p* < 0.01) (Figure 7A) and VEGF-A significantly decreased (*p* < 0.05) (Figure 7B) in excised mice tumours after treatment with liposome-loaded miR-34b when compared to liposome-loaded miR-1 and empty liposome treated group. Altogether, liposome-loaded miR-34b induced inhibition of the growth of anaplastic thyroid carcinoma in mouse xenograft models. 

## 4. Discussion

In the present study, miR-34b significantly increased in metastatic anaplastic thyroid carcinoma cells (from the lymph node metastases, BHT-101) when compared to the primary anaplastic thyroid carcinoma cells (8505C) following liposome-loaded miR-34b transfection (Figure 1(AI)) and no significant changes observed for p53 (Figure 1(AII)). Also, VEGF-A was shown to be more downregulated in response to miR-34b overexpression in comparison with other related genes demonstrating that the correlation between miR-34b, as the downstream regulator of p53, and p53 is more complicated than originally thought. Our functional studies also indicated that carcinoma cells in BHT-101 were responsive to miR-34b manipulation. Thus, BHT-101 cells were selected for the animal study. Notably, the level of miR-34b expression in BHT-101 generated-tumour showed a significant increase following intravenous delivery of miR-34b (Figure 7A). These results are also inconsistent with our previous results as well as results from other groups [8,9,10]. 

Our current delivery method indicated a successful targeted RNA delivery system with advantages over the RNA mimic delivery system in in vitro and in vivo. We have reported that miR-34b expression inversely regulated VEGF-A expression level in thyroid carcinoma [10]. The current study further showed the regulatory roles of miR-34b on VEGF-A expression. Transfection of miR-34b showed that overexpression of miR-34b significantly decreased VEGF-A expression at protein level in thyroid carcinoma cells and mice xenografts (Figure 1B and Figure 7B) as well as VEGF-A secretion levels in cell cultured media (Figure 1C). In addition, we noted a low vascularization in tumour sections in mouse receiving miR-34b on morphological examination. From these observations, miR-34b may affect angiogenesis in the thyroid carcinoma. It could regulate endothelial cell proliferation by decreasing VEGF-A secretion in the extracellular matrix. 

Involvement of VEGF-A with other proliferation regulatory genes such as Notch1 and Bcl-2 has been reported in anaplastic thyroid carcinoma [10]. Furthermore, Notch signalling directly or indirectly implicated in the regulation of VEGF-A. Our previous results also showed correlations between these three genes with miR-34b in the control of angiogenesis in thyroid carcinoma. Furthermore, Notch1 (Notch homolog 1) and Bcl-2 (B-cell lymphoma 2) are important in the regulation of cell proliferation. The inhibition of cancer cell proliferation following miR-34b overexpression occurred in other cancers [8,9]. Therefore, the anti-proliferative role of miR-34b in thyroid carcinoma is consistent with the earlier investigations and demonstrates the regulatory functions of miR-34b in different type of cancers. 

VEGF-A could play a more important role in the progression of thyroid carcinoma through angiogenesis process than Notch1 or Bcl-2. It is worth noting that Notch1 could be an oncogene or a tumour suppressor in context dependent manner [35]. VEGF-A, beside its role in angiogenesis, has been observed to be a survival factor for endothelial cells and tumour cell. It prevents apoptosis by inducing Bcl-2 expression [36]. 

Our results showed that miR-34b reduced cell proliferation on day 3 of transfection (Figure 2) and cell migration (Figure 3). In addition, miR-34b induced cell cycle arrest by accumulation of cells in G0-G1 phase and blocking of their entry into the S transitional phase and increased total apoptosis in anaplastic thyroid carcinoma cells (Figure 4). The reduced tumour volume observed in the animal study following intravenous delivery of miR-34b (Figure 5A–D) further confirmed the role of miR-34b. In addition to reduced tumour volume, tumour cell proliferation was significantly decreased following miR-34b-liposome delivered via intravenous route. The decrease in Ki-67 proliferative index and the histological studies confirmed the effect of the miR-34b in the in vivo analysis (Figure 6). Therefore, there may be an escape mechanism from therapeutic action within miR-34b network. For instance, miR-449 shares identical seed sequences and secondary structures with miR-34b. This could form a feedback network with p53 and E2F transcription factors and act as a possible escape mechanism for miR-34b [37]. Thus, further studies are required to investigate the effectiveness of the treatment. 

In conclusion: the present study demonstrated that liposome-loaded miR-34b proficiently delivered to anaplastic thyroid carcinoma cells both in vitro and in vivo. This delivery strategy could inhibit cancer cell proliferation, migration and angiogenesis in vitro and reduce tumour size and proliferation in xenotransplantation mouse model. To our knowledge, this is the first report of systemic delivery of miR-34b using a targeted RNA delivery system in anaplastic thyroid carcinoma. This is of clinical relevance as there is an absence of effective treatment options for this highly lethal cancer.

## Figures and Tables

**Table 1:** Characterization of miR-34b-5p-loaded PEGylated lipid particles.

Size (nm) ^a^	135.3 ± 10.80
Polydispersity index	0.311 ± 0.06
Zeta potential (mV)	39.16 ± 0.451
miR-34b-5p entrapment efficiency (%)	96.9 ± 2.18

The results indicated that the prepared liposome-loaded miR-34b liposomes have favourable characteristics for effective delivery of miR-34b such as high entrapment efficiency and size below 200 nm. Each sample contained 40 μg miR-34b in 300 μL isotonic sucrose solution. Three batches of HFDM liposomes were analysed (*n* = 3). ^a^ Size represents Z_ave_ ± SD as measured by Malvern Nano Zetasizer.

## Abbreviations

microRNA-34b-5p (miR-34b), hydration-of-freeze-dried-matrix (HFDM), vascular endothelial growth factor (VEGF), polyethylene glycol (PEG), *N*-[1-(2,3-dioleoyloxy)propyl]-*N*,*N*,*N*-trimethylammonium methyl-sulfate (DOTAP), 1,2-dioleoyl-sn-glycero-3-phosphoethanolamine (DOPE), 8505C (derived from anaplastic thyroid carcinoma) and BHT-101 (derived from lymph node with metastatic anaplastic thyroid carcinoma), Roswell Park Memorial Institute medium (RPMI 1640), Dul- becco’s Modified Eagle’s medium (DMEM), polyvinylidene difluoride (PVDF), TBST (Tris-buffered saline Tween 20: 120 mmol/l Tris–HCl, pH 7.4, 150 mmol/L sodium chloride, and 0.05% Tween 20), horseradish peroxidase (HRP), Cell Counting Kit-8 (CCK-8), phosphate-buffered saline (PBS), propidium iodide (PI), subcutaneous (s.c.), Enzyme-linked immunosorbent (ELISA), Notch1 (Notch homolog 1) and Bcl-2 (B-cell lymphoma 2).
